# Deletion of Histone Deacetylase 7 in Osteoclasts Decreases Bone Mass in Mice by Interactions with MITF

**DOI:** 10.1371/journal.pone.0123843

**Published:** 2015-04-15

**Authors:** Melissa Stemig, Kristina Astelford, Ann Emery, Jangyeun J. Cho, Ben Allen, Tsang-hai Huang, Rajaram Gopalakrishnan, Kim C. Mansky, Eric D. Jensen

**Affiliations:** 1 Department of Diagnostic and Biological Sciences, University of Minnesota, Minneapolis, Minnesota, United States of America; 2 Department of Developmental and Surgical Sciences, University of Minnesota, Minneapolis, Minnesota, United States of America; 3 Institute of Physical Education, Health and Leisure Studies, National Cheng Kung University, Tainan City, 701, Taiwan; University of Oulu, FINLAND

## Abstract

Molecular regulators of osteoclast formation and function are an important area of research due to the central role of osteoclasts in bone resorption. Transcription factors such as MITF are essential for osteoclast generation by regulating expression of the genes required for cellular differentiation and resorptive function. We recently reported that histone deacetylase 7 (HDAC7) binds to and represses the transcriptional activity of MITF in osteoclasts, and that loss of HDAC7 in vitro accelerated osteoclastogenesis. In the current study, we extend this initial observation by showing that conditional deletion of HDAC7 in osteoclasts of mice leads to an in vivo enhancement in osteoclast formation, associated with increased bone resorption and lower bone mass. Expression of multiple MITF target genes is increased in bone marrow derived osteoclast cultures from the HDAC7 knockout mice. Interestingly, multiple regions of the HDAC7 amino-terminus can bind to MITF or exert repressive activity. Moreover, mutation or deletion of the HDAC7 conserved deacetylase catalytic domain had little effect on repressive function. These observations identify HDAC7 in osteoclasts as an important molecular regulator of MITF activity and bone homeostasis, but also highlight a gap in our understanding of exactly how HDAC7 functions as a corepressor.

## Introduction

The formation and maintenance of the skeleton is conducted by two cell types- osteoblasts, which build bone, and osteoclasts, which resorb or degrade bone. Carefully coordinated local and systemic changes to their relative activities are responsible for increased bone formation during physiological bone growth and modeling, and proper responses to mechanical stresses or trauma. In contrast, unbalanced osteoclast formation or activity leads to pathological bone loss in many conditions. Thus, understanding the molecular regulators that control osteoclast differentiation or resorptive function is important in the development of new diagnostic or therapeutic strategies to manage pathological bone destruction.

Osteoclast differentiation is influenced by a multitude of cytokines, most significantly M-CSF (macrophage colony stimulating factor) and RANKL (receptor activator of NF-kappa B ligand) [1]. Both RANKL and M-CSF stimulation have been shown to regulate numerous transcription factors required for osteoclast differentiation including MITF, PU.1, c-Fos and NFATc1. These factors act on myeloid precursors of the monocyte/macrophage lineage to induce specification and commitment to the osteoclast cell fate, and mediate cell-cell fusion into large multinucleated polykaryons. They further activate a characteristic program of cell-substrate interactions and produce a number of bone degrading enzymes to create a highly acidified microenvironment that breaks down the mineral and organic components of the underlying bone matrix.

MITF (microphthalmia associated transcription factor) is a basic helix loop helix transcription factor that along with its partner, PU.1, has been shown to regulate genes necessary for osteoclast differentiation[2–6]. In work published by Sharma et al., they demonstrated that MITF and PU.1 could be detected at both the *Acp5* and *Ctsk* promoters in osteoclast precursors stimulated only with M-CSF, conditions under which neither gene is expressed[5]. Upon co-stimulation with M-CSF and RANKL to initiate osteoclastic differentiation and activation of these promoters, they demonstrated co-recruitment of the SWI/SNF chromatin remodeling complex, activated p38 MAP kinase, and subsequent recruitment of NFATc1 to these promoters. The presence of MITF at the *Acp5* and *Ctsk* promoters prior to active gene expression suggested that transcriptional activation might be repressed prior to the initiation of differentiation.

This suggestion led us to examine the ability of MITF to interact with histone deacetylase (HDAC) corepressors. We previously reported that MITF interacts with and is functionally repressed by HDAC7[7]. Knockdown of HDAC7 expression by shRNA leads to an enhancement of M-CSF/RANKL stimulated osteoclastogenesis in vitro. Moreover, MITF/HDAC7 interaction was disrupted by RANKL stimulation, suggesting that loss of HDAC7’s repression of MITF might contribute to RANKL induced gene expression and osteoclast differentiation. Subsequent to our initial report, Jin and colleagues published data that confirmed HDAC7’s action as an inhibitor of osteoclasts, both in vitro and in a mouse conditional knockout system[8]. Their data suggest an inhibitory mechanism in which HDAC7 inhibits osteoclastogenesis through alterations in NFATc1, β-catenin and Cyclin D1.

The goal of the current study is to investigate further the function of HDAC7 and its interactions with MITF in osteoclasts. In a continuation of our previous study using *Hdac7* shRNAs in osteoclast cultures, we characterize the in vivo bone phenotype of mice conditionally deleted for HDAC7 in osteoclasts. Significantly, these mice exhibit a progressive loss of bone mass due to an increase in osteoclast formation and enhanced bone resorption. Toward further testing the importance of MITF-HDAC7 interaction, we found that suppression of MITF in cultures of HDAC7 knockout osteoclasts largely corrected the heightened osteoclast formation caused by HDAC7 deletion. These data give biological significance to our previously described in vitro interaction between HDAC7 and MITF [7]. We used co-immunoprecipitations to identify more specifically a region of HDAC7 involved in physical binding to MITF. Interestingly, multiple regions of HDAC7 displayed repressive activity towards MITF. Together this study reinforces HDAC7’s importance as an in vivo regulator of osteoclastogenesis achieved by its negative regulation of MITF and extends our previously published in vitro study [7].

## Materials and Methods

### Breeding of *HDAC7* conditional knockout mice

*Hdac7* floxed mice were obtained from Dr. Eric Olson (UT Southwestern Medical Center) in a mixed background of C57BL/6 and 129Sv as described in Chang et al. [9]. These mice were crossed with B6.129-*Lyzstm1(cre)Ifo*/J mice (*LysM-Cre)* in a C57BL/6 background, which expresses Cre recombinase in cells of the myeloid lineage (Jackson Labs). *Hdac7* ^*flox/*^ *+; LysM-Cre* males and *Hdac7* ^*flox*^/+ females from the initial cross or from subsequent generations were then mated to generate *+/+; LysM-Cre* mice designated as wild type (WT), *Hdac7* ^*flox*^/+; *LysM-Cre* designated as conditional heterozygotes (HET) and *Hdac7* ^*flox*^/HDAC7^*flox*^; *LysM-Cre* designated as conditional knockouts (KO). These *Hdac7* ^*flox*^ *; LysM-Cre* conditional mice were used for the in vivo and ex vivo experiments presented in Figs 2 through 6. *Hdac7* ^*flox*^ mice were crossed to *c-fms-Cre* mice obtained from Dr. Jeffrey Pollard (Albert Einstein College of Medicine of Yeshiva University) using a similar breeding strategy as outlined above to generate *Hdac7* ^*flox*^; c-*fms-Cre* mice for bone marrow macrophage cultures shown in S1 Fig.

### Ethics

The use and care of these mice was reviewed and approved by the University of Minnesota Institutional Animal Care and Use Committee, IACUC protocol number 1208A18545. Isolation and culture of osteoclasts from mouse bone marrow cells, as well as virus generation and viral transduction of osteoclasts were performed under approval of the University of Minnesota Institutional Biosafety Committee, permit number 1207H17362.

### *In vivo* Analyses

#### Micro-CT Analysis

μCT was performed at the University of Minnesota. Femora were subjected to μCT scanning (model XT H 225, Nikon Metrology, Inc.) at conditions of 90 kV, 90 μA, 0.5° steps with 708 ms exposure time and pixel size at 12 μm. 3-D reconstructions were made from the original images using the software CT Pro 3D (Nikon metrology). VG Studio Max 2.1 (Volume Graphics GmbH) was used for visualization and 3-D rendering and creation of. bmp files. Then, histomorphometry analyses were performed using SkyScan CT-Analyzer Version 1.12 (Bruker microCT).

#### Dynamic Histomorphometry

50 mg/kg of tetracycline (Sigma-Aldrich) was delivered to mice by intraperitoneal injection 7 days and 2 days prior to harvest. Femora were dehydrated in gradient alcohol, cleared with xylene, and embedded in methylmethacrylate for dynamic histomorphometric analysis. Serial frontal sections 5 μm in thickness of each sample were made using a hard tissue microtome (Polycut E, Leica Microsystems). Photographs were taken by using a digital charged coupled device (CCD) camera (DP71, Olympus) attached to a fluorescent microscope with a long pass filter (Model: 41012, Chroma Technology Corporation). Lengths of fluorescent labeled/non-labeled measurement and distances between doubling were done by using the software Image Pro Plus 6.1 (Media Cybernetics). Secondary spongiosa 1–3 mm below the growth plate in the metaphysis of the distal femur was subjected to dynamic histomorphometric analyses according to the recommendations of the ASBMR Histomorphometry Nomenclature Committee[10].

#### ELISA

Serum was harvested from animals at 3 months of age and subjected to ELISA as per manufacturer’s protocol. CTX was detected using the RatLaps EIA (IDS). Osteocalcin was detected using a Mouse Osteocalcin Kit (Biomedical Technologies Inc.)

### *In vitro* analysis

#### Harvest and culture of primary osteoclasts

Primary bone marrow macrophages were harvested from the femurs and tibiae of 4-week-old WT, HET and KO mice. The femurs and tibiae were dissected and adherent tissue was removed. The ends of the bones were cut and the marrow was flushed from the inner compartments. Red blood cells were lysed from the flushed bone marrow tissue with RBC lysis buffer (150 mM NH_4_Cl, 10 mM KHCO_3_, 0.1 mM EDTA, pH7.4) and the remaining cells were plated and cultured overnight in 100 mm tissue culture dishes (Corning) in osteoclast media (phenol red-free alpha-MEM (Gibco) with 5% heat-inactivated fetal bovine serum (Hyclone), 25 units/mL penicillin/streptomycin (Invitrogen), 400 mM L-Glutamine (Invitrogen), and supplemented with 1% CMG 14–12 culture supernatant[11] containing 1.2 μg/mL M-CSF). The non-adherent cell population, including osteoclast precursor cells, was then separated and re-plated in 24-well plates (Corning) at 1.7x10^4^ cells/cm^2^ in osteoclast media supplemented with 1% CMG 14–12 culture supernatant. Two days later cells were refed with 1% CMG 14–12 culture supernatant and 30 ng/mL RANKL (R&D Systems) to stimulate osteoclast differentiation. Osteoclast activity was assayed on calcium phosphate plates (Corning). The demineralized area was photographed by light microscopy and then analyzed using NIH Image J.

#### TRAP Stain

Primary osteoclasts were fixed with 4% paraformaldehyde and washed with PBS. The cells were then stained for tartrate resistant acid phosphatase (TRAP) expression with using Naphthol AS-MX phosphate and Fast Violet LB salt according to the protocol provided in BD Biosciences Technical Bulletin #445. Cells were then imaged and photographed with light microscopy and the measurements were analyzed using NIH Image J.

#### Viral Transfection

Bone marrow macrophages were isolated as described above. Prior to stimulation with RANKL, the cells were incubated with 100 MOI of adenovirus (EGFP control, CRE recombinase, HDAC7 wild type or HDAC7^D692A/694A^) for 3 hours at 37°C in the presence of 1% CMG 14–12 culture supernatant. After 3 hours, the media containing adenovirus was removed and cells were fed with 1% CMG 14–12 culture supernatant and RANKL (30 ng/mL, R&D Systems). After five days, RNA was extracted for use in qRT-PCR, protein was extracted for western blotting, or cells were stained for TRAP.

Lentiviral vectors encoding shRNAs against *Mitf* (Open Biosystems) or a control shRNA were used to produce replication defective lentivirus according to the manufacturer’s protocols. Viral stocks were titrated by infection in HeLa cells. Bone marrow macrophages were isolated as described above. Forty-eight hours after M-CSF stimulation, lentiviruses were added and incubated for 18 hours at 37°C in the presence of 1% CMG 14–12 culture supernatant. The lentivirus-containing supernant was then removed and cells were fed with 1% CMG 14–12 culture supernatant and RANKL (30 ng/mL, R&D Systems). After five days, RNA was extracted for use in qRT-PCR or cells were stained for TRAP.

#### Real-time qRT-PCR

RNA was harvested from cells plated in triplicate using Trizol Reagent (Ambion, Life Technologies) and quantified using UV spectroscopy. cDNA was then prepared from 1 μg RNA using the iScript cDNA Synthesis Kit (Bio-Rad) as per the manufacturer’s protocol. Quantitative real-time PCR was performed in duplicate using the MyiQ Single Color Real-Time PCR Detection System (Bio-Rad). Each 20 μl reaction contained 1 μl cDNA, 10 μl iTaq Universal Sybr Green Supermix and 500 nM forward and reverse primers. The PCR conditions were as follows: 95°C for 3 minutes, and the 40 cycles of 94°C for 15 seconds, 56°C for 30 seconds and 72°C for 30 seconds, followed by melting curve analysis (95°C for 5 sec, 65°C for 5 sec and then 65°C to 95°C with 0.5°C increase every 5 seconds). Experimental genes were normalized to *L4*. Primer sequences are listed in S1 Table.

#### Immunoblotting

Cell protein lysates were harvested from osteoclasts grown in 12 well dishes (Corning) in modified RIPA buffer (50 mM Tris pH 7.4, 150 mM NaCl, 1% IGEPAL, 0.25% sodium deoxycholate, 1 mM EDTA) supplemented with Halt Protease & Phosphatase Inhibitor Cocktail (Thermo Scientific). Lysates were cleared by centrifugation at 12,000Xg at 4°C. Proteins were resolved by SDS-PAGE, transferred to PVDF membrane (Millipore), subjected to western blotting by standard protocols, and visualized using ECL Prime (G.E. Health Systems). Blots were blocked and incubated at 4°C with primary antibodies diluted in TBS/0.1% Tween-20 plus 3% bovine serum albumin. Primary antibodies used were HDAC7-Abcam (Ab12174) 1:2000 dilution; ^α^-TUBULIN- Santa Cruz (SC-5546) 1:1000 dilution; MYC- Santa Cruz (SC-40) 1:1000 dilution; MITF- Abcam (Ab12039) 1:1000 dilution; ACTIN- Santa Cruz (SC-1616) 1:2,000 dilution; FLAG—Sigma-Aldrich (F1804) 1:5,000 dilution. Horseradish-peroxidase conjugated secondary antibodies were diluted in TBS/0.1% Tween-20 plus 5% nonfat dry milk. Secondary antibodies used were from G.E. Health Systems: Amersham ECL anti-mouse (NA-931) and anti-rabbit (NA-934) at 1:8000, or Santa Cruz: anti-goat (SC-2020) at 1:12,000 dilution.

#### Co-immunoprecipitations

293T cells were maintained in Dulbecco’s modified Eagle medium supplemented with 10% bovine calf serum, 2% L-glutamine and 0.5% penicillin/streptomycin. Cells were seeded in 60 mm plates (Corning) at 1.3X10^4^ cells/cm^2^ density and transfected by Lipofectamine Plus Reagent (Invitrogen). The vector encoding FLAG-tagged MITF was reported by Bronisz et al.[12] The HDAC7 full length and deletion constructs previously described [13] were excised and ligated into the pCMV-3Tag-2A plasmid (Agilent) by an EcoR1/Xho1 digestion to give an N-terminal myc tag and were verified by restriction digestion and DNA sequencing. 5 μg of each plasmid were transfected using 12 μL of Lipofectamine and 8 μL of Plus per dish according to the manufacturer’s recommended protocol. Twenty-four hours after transfection, cells were harvested in NP-40 lysis buffer (20 mM Tris, pH 8.0, 137 mM NaCl, 10% glycerol, 1% NP-40 and protease and phosphatase inhibitors). Extracts were incubated with target antibody and Protein A/G beads (Pierce) overnight at 4°C. The following day, immunoprecipitates were precipitated and washed three times in lysis buffer. Bound proteins were resuspended in sample buffer and resolved by SDS-PAGE. The resolved proteins were transferred to PVDF membrane, blocked and blotted in primary antibody overnight at 4°C. The next day the blot was incubated with horseradish peroxidase anti-rabbit or anti-mouse for one hour at room temperature. Antibody binding was detected using ECL system (GE Healthcare). For immunoblotting conditions and antibody concentrations see Immunoblotting section above.

#### Luciferase Assay

NIH 3T3 were seeded at 1X10^3^ cells/cm^2^ density in 12-well plates (Corning) and transiently transfected with 2 μL of Lipofectamine and 4 μL of Plus Reagent (Invitrogen). 300 ng gal-UAS luciferase reporter, 1 μg pMI-GAL4-MITF, 500 ng full length or deletion constructs of myc-tagged HDAC7 were used in the transfections. Transfections were done in serum-free media for 3 hours and then media containing serum was added back to the transfection. Each transfection was performed in triplicate wells. Twenty-four hours after transfection, cells were washed with PBS and lysed with 100 μL Cell Culture Lysis Reagent (Promega). Luciferase activities were measured using a GloMax20/20 Luminometer (Promega) using the Luciferase Assay System (Promega) according to the instructions of the manufacturer, and were normalized to total protein content, which was measured using the DC Protein Assay (Bio-Rad) and measured against a standard curve of known BSA concentrations.

#### Statistical Analysis

All experiments were run in triplicate, performed at least 3 times, and results are expressed as mean +standard deviation. Student’s *t*-test or a one-way ANOVA analysis followed by a Tukey’s multiple comparison test were used to compare data using GraphPad Prism version 5 for Mac OS X.

## Results

### HDAC7 Inhibits Osteoclast Differentiation

**Fig 1:**
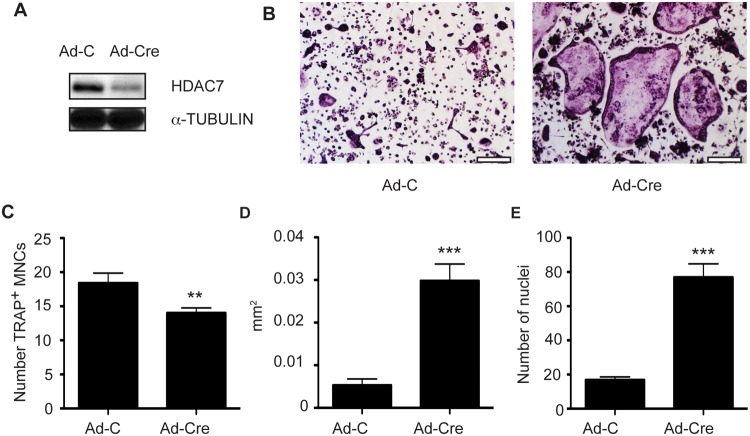
Adenoviral-CRE mediated *Hdac7* deletion enhances osteoclast formation. Bone marrow macrophage cultures from *Hdac7* ^*flox*^/*Hdac7* ^*flox*^ mice were infected either with control adenovirus encoding EGFP (Ad-C) or encoding CRE recombinase (Ad-Cre). Western blotting (A) indicates a decrease in HDAC7 protein levels. Osteoclasts were differentiated by stimulation with M-CSF and RANKL for 5 days and visualized by TRAP staining (B). Scale bar 200 μm. (C-E) Quantitation of the number (C), average size (D), and average number of nuclei (E) in TRAP-positive multinucleated cells. Experiments were performed at least three times in triplicate. Samples were compared using an unpaired Student’s t-test ** p<0.01 vs. Ad-C, *** p<0.0001 vs. Ad-C.

In order to confirm our recent finding that HDAC7 acts as an inhibitor of osteoclast differentiation in culture, we compared osteoclast differentiation of bone marrow macrophages (BMMs) from *Hdac7* ^flox^/*Hdac7* ^flox^ mice which were infected either with control adenovirus expressing EGFP (Ad-C) or CRE recombinase (Ad-Cre). By western blot, HDAC7 expression was reduced in the osteoclasts infected with Ad-Cre (Fig 1A). As shown in Fig 1B–1E, osteoclasts formed in cultures infected with Ad-Cre are larger and have more nuclei per osteoclast compared to Ad-C infected cultures. The average size of TRAP-positive multinucleated cells containing 3 or more nuclei was increased by approximately six-fold (Fig 1D) while the average number of nuclei per multinucleated cell increased by about 4.5-fold to nearly 76 nuclei per cell (Fig 1E). Consistent with our previous studies, we noted a decrease of approximately 24% in total number of TRAP positive multinucleated cells (MNCs, Fig 1C) following Ad-Cre transduction. We attribute this decrease in overall number of TRAP-positive cells to increased cellular fusion; multiple individual cells fusing together will decrease the total number of cells while increasing their size and number of nuclei.

### *Hdac7* KO Mice Are Osteopenic

**Fig 2:**
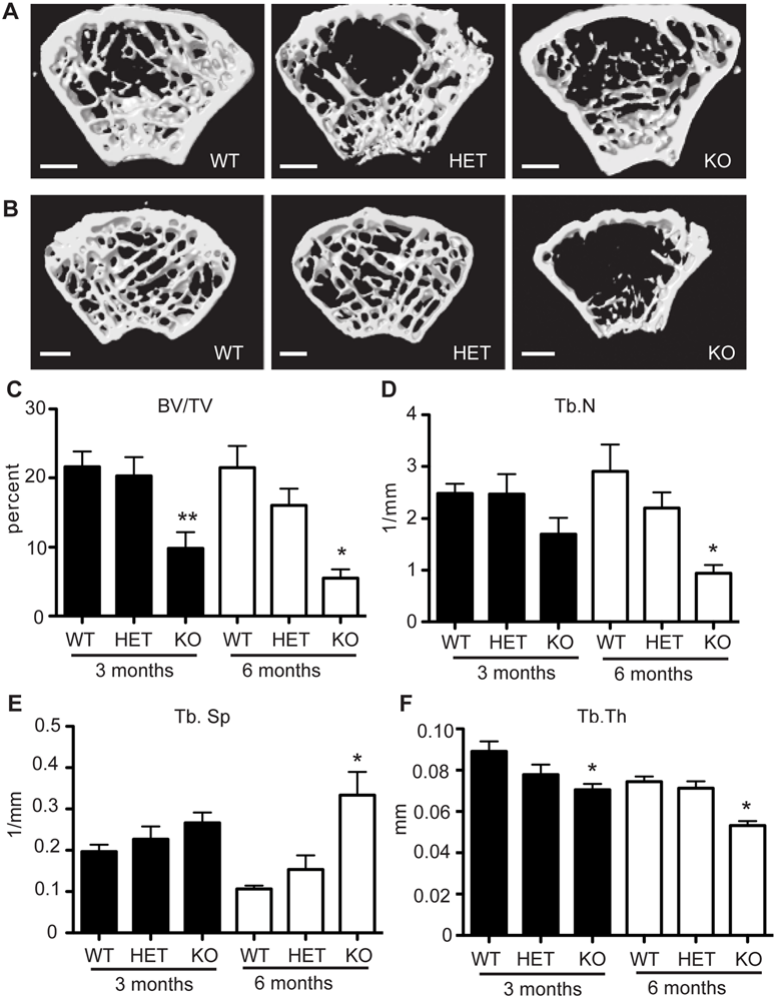
MicroCT analysis of Hdac7 conditional knockout bones. Representative μCT scans of distal femur from *Hdac7* WT, HET and KO mice at (A) 3 months and (B) 6 months age. Comparison of bone volume/ total volume (C), trabecular number (D), trabecular separation (E) and trabecular thickness (F) at 3 months (black bars) and 6 months (white bars). Data represent the mean values of 8 WT, 7 HET, and 9 KO at 3 months and 5 WT, 7 HET and 5 KO at 6 months. Scale bar 500 μm. Samples were compared using a one way ANOVA followed by a Tukey’s post test * p<0.05 vs. respective WT. ** p<0.01 vs. WT.

To evaluate the skeletal phenotype of mice that are null for HDAC7 expression in the osteoclast lineage, we bred *Hdac7* floxed mice with *LysM*-Cre mice. For the remainder of the paper, *+/+; LysM-Cre* will be referred to as wild type (WT), *Hdac7* ^*flox*^ */+; LysM-Cre* as heterozygotes (Het), and *Hdac7* ^*flox*^ */Hdac7* ^*flox*^ *; LysM-Cre* as knockout (KO). μCT analysis of 1-month-old animals revealed no significant difference in bone volume fraction between WT, HET and KO animals (data not shown). KO mice at 3 months of age had approximately half the trabecular bone volume at the distal femur compared to WT and HET mice (Fig 2A and 2C, black bars). This decrease in BV/TV was accompanied by trends towards reduced trabecular number (Fig 2D), increased trabecular separation (Fig 2E) and a significant reduction in trabecular thickness (Fig 2F). Bones from HET mice showed modest increases in trabecular separation and decreases in trabecular thickness at this time. To further characterize skeletal homeostasis during aging of the *Hdac7*; *LysM*-Cre mice, we performed micro-CT on the mice at 6-months of age (Fig 2B). At this age, KO mice had four times less bone volume compared to WT mice (Fig 2C, white bars) with further reductions in trabecular number and thickness (Fig 2D and 2F), and an increase in trabecular separation (Fig 2E). At 6 months, HET mice showed an apparent, although not statistically significant, trend towards reduced bone by each of these measures. These results indicate that loss of HDAC7 in osteoclasts is associated with a progressive reduction in trabecular bone.

### *Hdac7 KO* mice have enhanced osteoclast differentiation and activity

**Fig 3:**
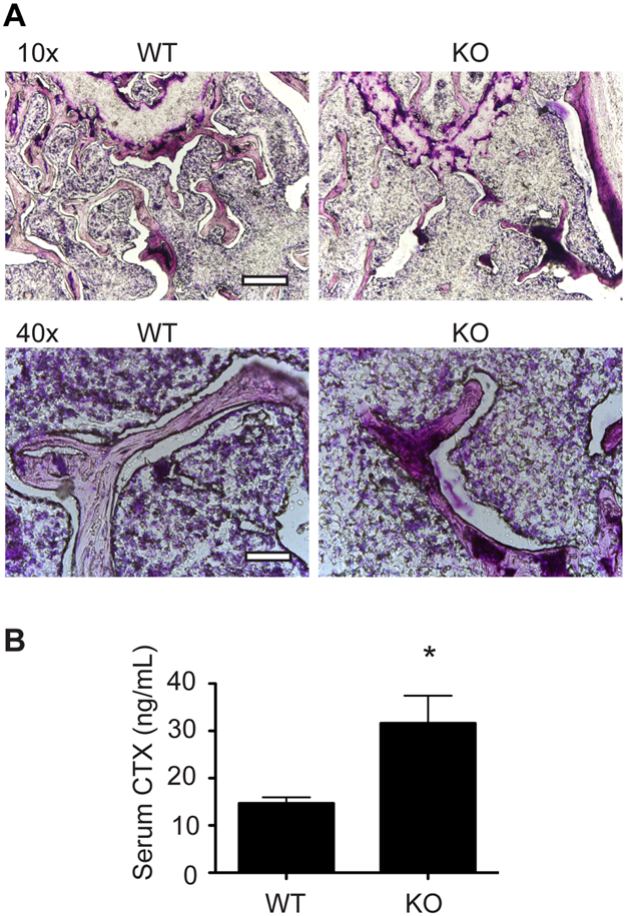
*Hdac7* KO bones show increased osteoclasts and bone resorption. (A) TRAP staining reveals increased osteoclasts in trabecular bone at the proximal tibia from KO mice compared to WT controls at 3 months age. Upper row at 10x magnification, scale bar 200 μm. Lower row at 40x magnification, scale bar 50 μm (B) Measurement of C-telopeptide of collagen (CTX) in serum of WT and KO mice at 3 months. Data represent the mean of 7 animals of each genotype. Samples were compared using an unpaired Student’s t-test * p<0.05 vs. WT.

**Fig 4:**
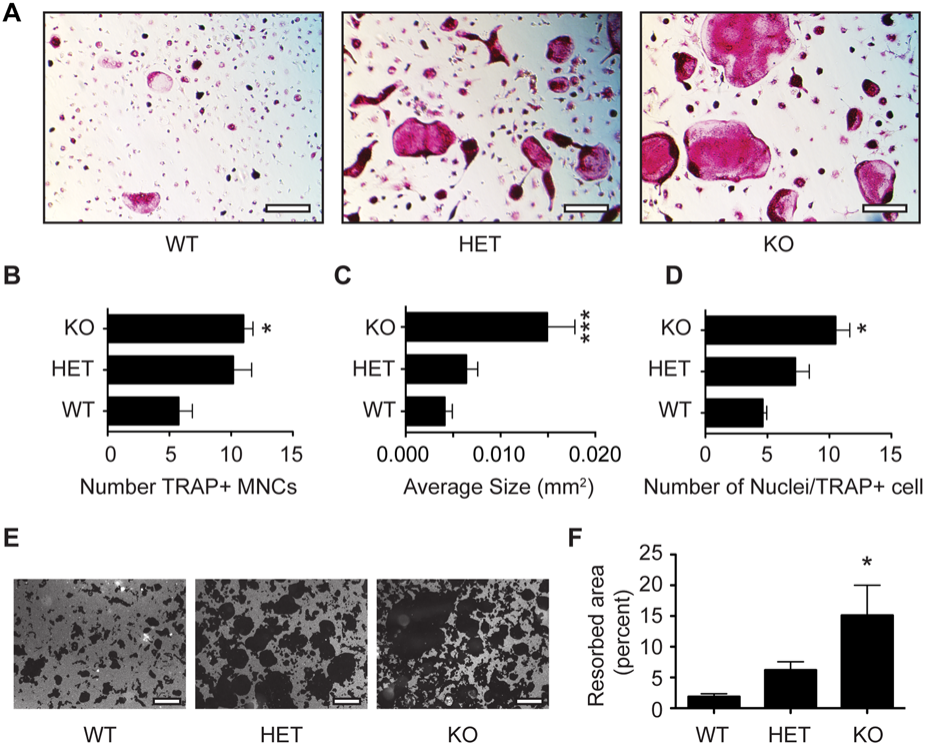
Osteoclast cultures from *Hdac7* WT, HET and KO mice. TRAP staining (A) reveals osteoclasts obtained from M-CSF and RANKL stimulation of BMMs cultured from *Hdac7* WT, HET and KO animals. (B) Number, (C) size and (D) number of nuclei per TRAP-positive multinucleated cells. Photographs (E) and quantitation (F) of resorption activity of WT, HET and KO osteoclast cultures grown on calcium phosphate surfaces. Experiments were performed at least three times in triplicate. In both (A) and (E) scale bar 200 μm. Samples were compared using a one way ANOVA followed by a Tukey’s post test* p<0.05 vs. WT ** p<0.01 vs. WT.

Alterations to bone mass or volume arise by changes to the net activities of bone formation and bone resorption. The increased osteoclast formation seen in our Ad-Cre studies shown in Fig 1 suggested that the reduced bone volume in *Hdac7* KO bones could be due to increased bone resorption. To test this hypothesis, we visualized osteoclasts in histological sections from WT and KO distal femurs by TRAP staining. The KO bones exhibited considerably more TRAP staining than WT controls (Fig 3A). In agreement with the μCT analysis, trabeculae in the KO bones appeared to be more sparse and thinner than WT. Consistent with this, assessment of resorption in WT and KO animals using CTX ELISA (Fig 3B) indicated that in vivo bone resorption in KO animals was doubled compared to WT. Moreover, when BMMs from these mice were cultured, the resulting TRAP positive multinucleated osteoclasts from KO cultures were more numerous, larger and had more nuclei per multinucleated cell than WT cultures (Fig 4A–4D). To measure activity of the cultured osteoclasts, BMMs from WT, HET and KO mice were cultured on calcium phosphate coated plates. KO osteoclasts produced significantly more demineralized area compared to either WT or HET osteoclasts (Fig 4E and 4F). To further confirm these data, we generated *Hdac7* ^*flox*^ */Hdac7* ^*flox*^ *; c-fms-Cre* mice and obtained BMM cultures from them. Similar to our observations of the *LysM-Cre* model, in vitro cultures of osteoclasts with *c-fms-Cre*-mediated deletion of *Hdac7* showed a modest reduction in overall number of TRAP-positive multinucleated (from 76 to 54 cells per field, p = 0.053), but a large increase in their average size (from 0.018 to 0.078 mm^2^, p = 0.0003) (S1 Fig). Together these data indicate that direct changes in the osteoclast lineage in *Hdac7* KO mice results in enhanced osteoclast function, leading to a progressive loss of bone mass.

We assessed the status of bone formation by performing dynamic histomorphometric analyses in WT, HET and KO mice to determine whether there were alterations to bone anabolism that might contribute to the low bone mass phenotype. Double tetracycline labeling revealed no significant changes to the bone formation rate, mineral apposition rate or mineralizing surface per bone surface (Table 1), indicating little alteration to osteoblast activity in these mice. As an additional measure of osteoblast activity, we measured serum osteocalcin levels from WT and KO animals by ELISA (HETs were not examined in this assay) and found no significant differences between WT and KO animals (Table 1). From these observations, we conclude that reduced bone formation does not contribute to the low bone mass of *Hdac7*; *LysM-Cre* mice

**Table 1:** Bone Formation Parameters for *Hdac7^flox^; LysM-Cre* mice.

Genotype	MS/BS	MAR μM/day	BFR μm^3^/μm^2^/day	Osteocalcin ELISA ng/mL
WT	0.23 ±0.11	2.06 ±0.01	0.65 ±0.19	35.6 ±2.7
HET	0.15 ±0.07	1.88 ±0.12	0.30 ±0.09	Not determined
KO	0.30 ±0.09	1.79 ±0.10	0.53 ±0.18	30.4 ±0.7

### Loss of HDAC7 enhances osteoclast differentiation by increased MITF activity

**Fig 5:**
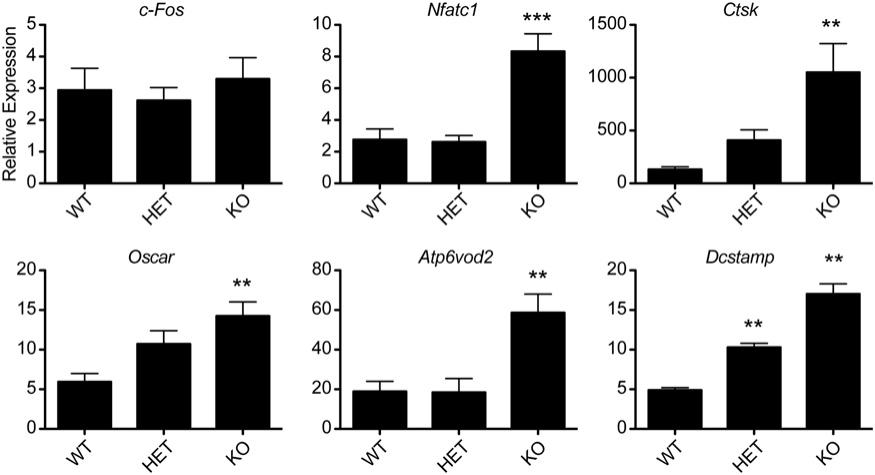
Gene expression of *Hdac7* WT, HET and KO osteoclast cultures. qRT-PCR comparing expression of osteoclast genes *c-Fos*, *Nfatc1*, *Ctsk*, *Oscar*, *ATP6v0d2* and *Dcstamp*. Data shown are the mean+SD of three independent experiments in which gene expression was measured from three wells of each genotype, with each PCR reaction performed in duplicate. Expression of each gene is graphed relative to *L4*. Samples were compared using a one way ANOVA followed by a Tukey post test ** p<0.01; *** p<0.001 vs. WT.

We next performed experiments to understand the mechanistic changes though which HDAC7 depletion in osteoclasts leads to low bone mass. We examined expression of genes important for osteoclast formation or function including *Dcstamp*, *Ctsk*, *Oscar* and *Atp6v0d2* in BMM-derived osteoclasts from the *Hdac7* KO animals. Expression of each of these genes, all known MITF targets, is upregulated 2–8 fold compared to BMMs from WT littermates (Fig 5). Other transcription factors besides MITF can regulate expression of *Dcstamp*, *Ctsk*, *Oscar* and *Atp6v0d2* so we also measured expression levels of other transcription factors whose occupancy of osteoclast promoters does not change with RANKL stimulation [5]. While *Nfatc1* expression was upregulated in *Hdac7* KO cells, *c-Fos* expression was not significantly changed.

**Fig 6:**
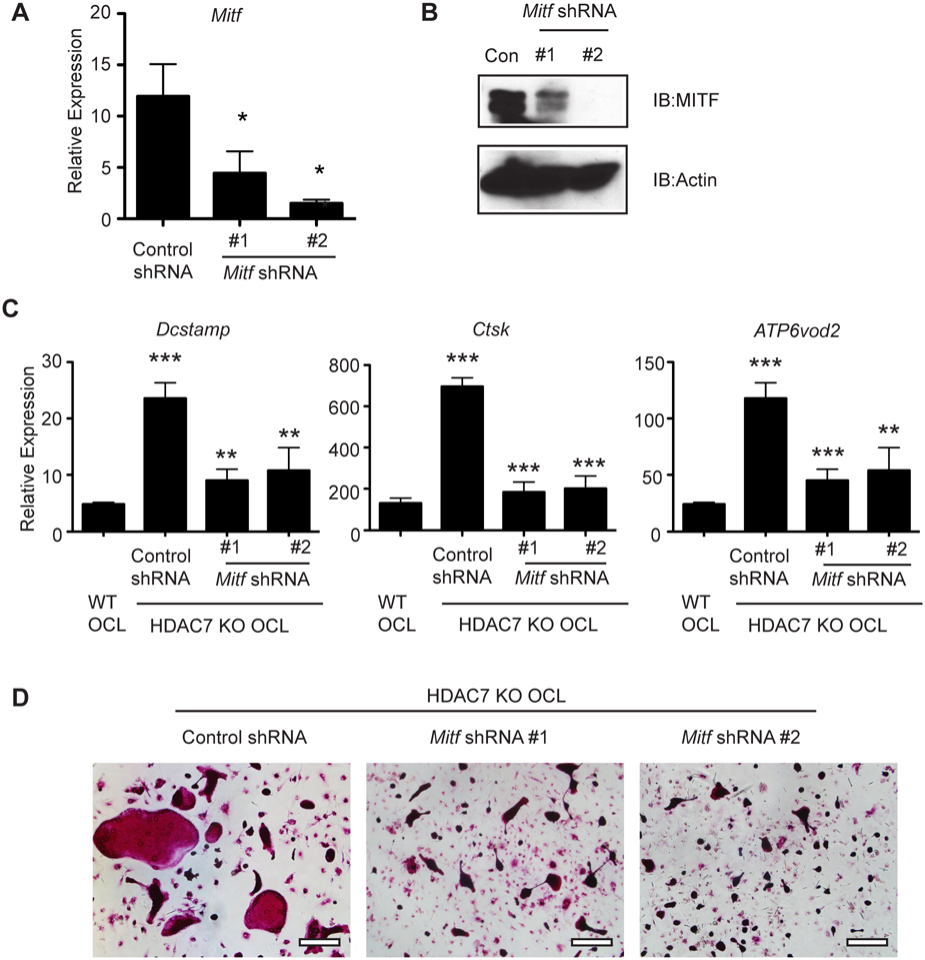
*Hdac7* KO in *Mitf* suppressed osteoclasts. qRT-PCR (A) and western blotting (B) in BMM cultures infected with lentivirus encoding a control shRNA or two distinct shRNAs against *Mitf*. (C) qRT-PCR measurement of *Dcstamp*, *Ctsk* and *Atp6v0d2* relative to *L4* in WT osteoclast cultures or *Hdac7* ^*flox*^ *; LysM-Cre* KO osteoclasts infected with lentivirus encoding control or *Mitf* shRNAs. (D) TRAP staining of osteoclasts from WT or KO osteoclasts infected with the indicated lentiviral shRNA vectors. Experiments were performed at least three times in triplicate. Scale bar 200 μm. Samples were compared using a one way ANOVA followed by a Tukey post test ** p<0.01, *** p<0.0001, comparing control shRNA to WT, and *Mitf* shRNAs to control shRNA.

Our previous work showing that HDAC7 acts as a corepressor for the MITF transcription factor in vitro, led us to hypothesize that *Hdac7* KO enhances in vivo osteoclast formation by de-repressing MITF activity in osteoclast precursors. From this model, we predicted that *Hdac7* KO should be unable to enhance osteoclast formation in cells deficient for MITF. Accordingly, we infected osteoclast progenitors from *Hdac7* KO animals with viruses encoding shRNA to *Mitf* or a control shRNA. As shown in Fig 6A and 6B, we detected reduced *Mitf* expression both at the RNA and protein level in the osteoclasts transduced with *Mitf* shRNAs. *Hdac7* KO cells infected with control shRNA showed increased expression of MITF gene targets such as *Dcstamp*, *Ctsk* and *Atp6v0d2* compared to wild type osteoclasts (Fig 6C). In contrast, *Hdac7* KO cells depleted for MITF exhibited levels of MITF targets similar wild type osteoclasts (Fig 6C). TRAP staining of these cultures showed a similar pattern, with *Mitf* shRNA attenuating the *Hdac7* KO phenotype (Fig 6D). Together, these results suggest that HDAC7 represses MITF activity to regulate the rate of osteoclast formation in vivo.

### A small region of the amino-terminal domain of HDAC7 binds to MITF

**Fig 7:**
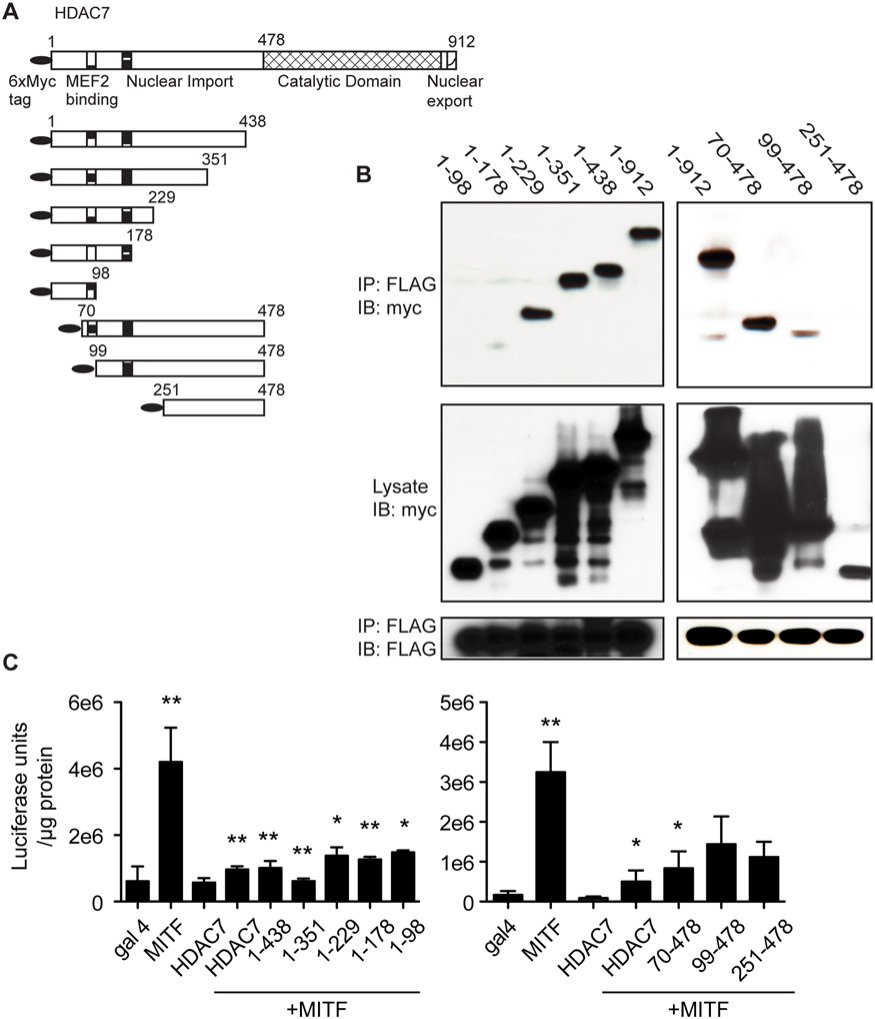
Interactions between MITF and HDAC7. (A) Schematic of myc-HDAC7 deletion constructs with amino acid numbers and HDAC7 known functional domains indicated. (B) 293T cells were transfected with plasmids encoding FLAG-MITF and the indicated myc-tagged HDAC7 constructs, subjected to FLAG immunoprecipitation and blotted against FLAG and myc. Co-immunoprecipitations were performed at least three times. (C) Activity of UAS-luciferase reporter construct in NIH 3T3 cells transfected with the indicated GAL4-MITF and myc-HDAC7 plasmids. Transfections were done in triplicate at least three times. For statistical analysis, MITF and HDAC7 alone are compared to the GAL4 control, while MITF+HDAC7 transfections are compared to GAL4-MITF alone. Samples were compared using a one way ANOVA followed by a Tukey post test * p<0.05 ** p<0.01.

In light of our data suggesting a functional interaction between HDAC7 and MITF in vivo, we sought to better understand their physical association. In our previous study, GAL-MITF amino acids 1–185 construct showed much more robust interaction with the HDAC7 N-terminus (aa 1–478) than C-terminus (aa 470–912) in co-immunoprecipitations[7]. To better define regions of the HDAC7 N-terminus that bind MITF, we tested a series of HDAC7 deletion constructs[13], shown schematically in Fig 7A, for their ability to interact with MITF. As shown in Fig 7B, myc-tagged full-length 1–912, 1–438, 1–351 and 1–229 HDAC7 were efficiently co-immunoprecipitated with FLAG-tagged MITF. However, we were unable to detect any substantial interaction between MITF and 1–178 or 1–98 of HDAC7. We were similarly able to detect interactions between MITF and HDAC7 aa 70–478 and 99–478, but not 251–478. These data indicate that HDAC7 binds MITF via a domain located between 178 and 250 of HDAC7.

To extend the physical interaction analyses to functional repression of MITF activity, we tested the ability of the HDAC7 deletion constructs to repress GAL4-MITF-dependent transcription of a UAS-reporter (Fig 7C). As expected, full-length HDAC7 was a strong repressor of GAL4-MITF activity. Unexpectedly, all fragments of HDAC7 tested in this assay displayed considerable repressive activity towards MITF. We observed no statistically significant differences in the luciferase activity obtained with MITF +full-length HDAC7 compared with MITF +HDAC7 deletion mutants.

### HDAC7 Inhibits Osteoclast Differentiation Independent of its Deacetylase Activity

**Fig 8:**
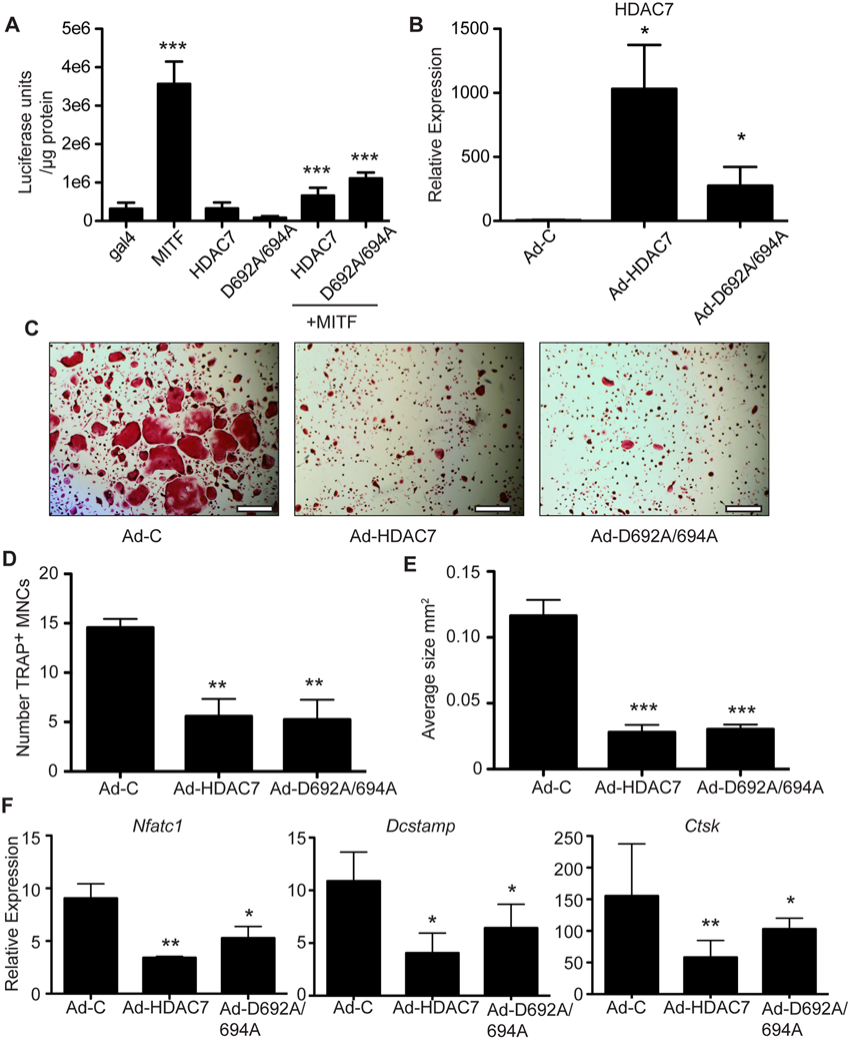
Repressive activity of HDAC7 deacetylase mutants. (A) Activity of UAS-luciferase reporter transfected with the indicated GAL4-MITF and myc-HDAC7 plasmids. For statistical analysis, MITF and HDAC7 alone are compared to GAL4 control, while MITF+HDAC7 transfections are compared to GAL4-MITF alone. Transfections were done in triplicate and performed at least three times. (B) qRT-PCR measurement of *Hdac7* expression in BMM cultures transduced with Ad-EGFP (Ad-C), Ad-HDAC7 or Ad-D692A/694A vectors, normalized to *L4* expression. (C) TRAP staining of BMM cultures transduced with adenoviral vectors and stimulated with M-CSF+RANKL for five days. Scale bar 500 μm. Measurement of the number (D) and average size (E) of TRAP-positive multinucleated cells from BMM cultures treated as in (C). (F) Osteoclast marker gene expression in BMM cultures treated as in (C). Experiments were done in triplicate at least three times. Samples were compared using a one way ANOVA followed by a Tukey post test * p<0.05, ** p<0.01, *** p<0.001 comparing Ad-Control to Ad-HDAC7 or Ad-D692A/694A

In previous studies, the broad spectrum deacetylase inhibitor TSA did not block HDAC7’s corepressor function towards MITF, suggesting that the deacetlyase activity of HDAC7 is not required to inhibit MITF activation of a reporter construct[7]. To further test whether the deacetylase activity of HDAC7 is necessary for its ability to inhibit MITF activation, we performed co-transfections with a UAS-luciferase reporter construct and GAL4-MITF, wild type HDAC7 or HDAC7 containing an inactivating mutation in the deacetlyase domain (D692A/694A)[14]. pMI-GAL4-MITF was able to activate the UAS-luciferase construct 10 fold while both wild type HDAC7 and D692A/694A were able to repress MITF activation, with no statistical difference between their repressive activity (Fig 8A). Since D692A/694A could inhibit MITF transcriptional activation, we next asked whether the deacetylase activity of HDAC7 is necessary for HDAC7 inhibition of osteoclast differentiation. We infected BMMs with an EGFP control adenoviral vector, adenovirus that overexpresses wild type HDAC7 or one that overexpresses D692A/694A. qRT-PCR indicated that both HDAC7 constructs were expressed to similar extents (Fig 8B). Like wild type HDAC7, D692A/694A inhibited osteoclast differentiation (Fig 8C–8E). Similarly, wild type HDAC7 and D692A/694A were able to inhibit osteoclast genes such as *Nfatc1*, *Dcstamp*, and *Ctsk* (Fig 8F). Taken together, we conclude that HDAC7’s deacetylase catalytic activity is not essential for its corepressor function.

## Discussion

The balance between proper skeletal homeostasis or pathological bone loss requires that the formation and function of osteoclasts be carefully regulated. Diseases such as osteoporosis, metastatic bone disease and arthritis all involve excessive osteoclast activity leading to bone destruction. This study provides compelling in vivo and in vitro evidence that osteoclast differentiation is regulated by HDAC7 acting as a repressor towards MITF, a transcription factor required for osteoclast differentiation. Consequently, deletion of HDAC7 in the osteoclast lineage leads to a progressive loss in bone mass due to exuberant osteoclast generation.

The importance of MITF as a regulator of osteoclasts has long been appreciated, starting with the mutant mouse *microphthalmia (Mitf* ^*mi*^ *)*, which exhibits severe osteopenia among other phenotypes [15]. Differentiation of osteoclasts from *Mitf* ^*mi*^ mice is defective, resulting in numerous TRAP-positive mononucleated cells that fail to undergo fusion into multinucleated osteoclasts and display perturbed ruffled borders. MITF is a basic helix-loop-helix leucine zipper class transcription factor. Its function as a transcriptional regulator is subject to multiple layers of regulation including phosphorylation[16,17], sumoylation[18] and interactions at target promoters with other transcription factors such as PU.1[2,5,6,19]. In early osteoclast progenitors stimulated with M-CSF only, MITF and PU.1 are recruited to and repress osteoclast gene promoters including *Ctsk* and *Acp5*[5,20]. Our current qRT-PCR data suggest that HDAC7 participates in regulation of these MITF downstream targets such as *Ctsk*, *Nfatc1*, *Dcstamp* and *Atp6v0d2* rather than on the upstream transcription factors such as c-Fos in progenitor cells. During further M-CSF and RANKL stimulated differentiation, MITF is phosphorylated by ERK1/2 and p38 MAP Kinases, leading to recruitment of coactivator proteins and transcriptional activation. MITF has been shown to be phosphorylated by both M-CSF, through ERK1/2 on serine 73, and by RANKL, through p38 MAPK on serine 307[21]. Phosphorylation of MITF enhances its ability to activate *Acp5*, *Oscar*[2] and *Dcstamp*[22] promoters by recruiting transcriptional coactivator protein complexes. It remains unclear whether these phosphorylation mechanisms regulate MITF/HDAC7 functional and physical interaction.

Little is known about the interplay between MITF and HDAC co-repressors, or the potential clinical significance of these interactions. Histone deacetylase inhibitors have been shown to inhibit osteoclast differentiation in culture [23–25], suggesting that deletion of HDACs might decrease osteoclast formation and increase bone mass. Indeed, HDAC inhibitors are under investigation as therapies for inflammatory osteocyte conditions such as arthritis and periodontitis (reviewed by [26]. Our previous findings with HDAC3 suppression were consistent with this paradigm, indicating that loss of HDAC3 reduces osteoclastogenesis[7]. In contrast, we unexpectedly found that suppression of HDAC7 enhanced M-CSF and RANKL induced osteoclast differentiation in culture[7], and the present in vivo studies further support this model, suggesting that HDAC7 is a negative regulator of osteoclast formation. Another recent report reached similar conclusions, confirming that HDAC7 overexpression blocked osteoclastogenesis in vitro, and showing that conditional knockout of HDAC7 decreased bone mass in vivo due to increased osteoclastic bone resorption, attributed to misregulation of NFATc1, β-Catenin, and Cyclin D1 [8]. From this, one might anticipate HDAC inhibition as deleterious to bone health. Indeed, there are some reports in the literature that indicate long-term administration of the HDAC inhibitor valproic acid as an anticonvulsant in epileptic patients is associated with increased fracture risk and reduced bone mineral density of uncertain etiology[8,27–29], although other studies found no significant association. Finally, work by McGee-Lawrence and colleagues showed that administration of the HDAC inhibitor SAHA to mice had no effect on osteoclastic function, but observed decreased bone mass attributed to impaired osteoblast differentiation [30]. These data clearly indicate a need for additional studies to better clarify the biological consequences of HDAC inhibition on osteoclasts and bone health.

Our deletion analysis reveals considerable complexity in the physical and functional interactions between MITF and HDAC7. We previously showed that both the HDAC7 N-terminus and C-terminus can repress MITF’s activity in luciferase reporter assays, and both the amino- and carboxyl- terminal fragments were co-immunoprecipitated with GAL-MITF, although the N-terminal fragment interacted more strongly than the C-terminus[7]. Co-immunoprecipitations between MITF and a set of HDAC7 deletion constructs indicated that the region of HDAC7 necessary for highest affinity binding resides between amino acids 178 and 229. We anticipated that the ability of the HDAC7 deletion fragments to bind to MITF would be requisite for their repression of MITF function. Surprisingly, all the HDAC7 N-terminal fragments tested in this assay displayed repressive activity towards MITF regardless of their physical association in co-immunoprecipitations. We envision several hypotheses that could account for these data. Multiple domains of HDAC7 might bind directly to MITF, HDAC7 and MITF might associate indirectly as part of a multi-protein complex, or some combination of direct and indirect interactions might be involved. We previously mapped similar complex interactions between HDAC7 and RUNX2[13], while deletion analysis of HDAC4 binding to the ANKRA transcription factor identified multiple direct and indirect protein-protein binding regions as well as an autoinhibitory domain of HDAC4 that impaired their binding[31].

The repressive activity displayed by the HDAC7 deletion fragments also raises the issue of exactly how does HDAC7 function as an MITF corepressor. All of the deletion fragments tested completely lacked the deacetylase catalytic domain, which is located in the carboxyl-terminal half of the HDAC7 protein, indicating that the catalytic domain is dispensable for repression. That conclusion is further demonstrated in Fig 8, in which full-length HDAC7 containing inactivating point mutations in the deacetylase catalytic domain shows similar repressive function towards osteoclast differentiation and gene expression to the wild type protein. Repression by HDAC7 that is insensitive to HDAC inhibitors was observed in multiple reports[7,32–35], and in many cases the C-terminal deacetylase catalytic domain is dispensable for repressive activity[7,13,36–38]. At least three repression domains in HDAC7 have been previously identified [36]. The mechanisms of this non-deacetylase mediated repression remain poorly understood. Some potential scenarios include impaired access of transcriptional activator complexes to MITF at target promoters, sequestration of MITF to transcriptionally repressive subnuclear domains, or recruitment by HDAC7 of non-deacetylase corepressors.

## Conclusions

In conclusion, in this report we present in vivo and in vitro evidence showing that extent of osteoclast formation and associations between the MITF transcription factor and HDAC7 transcriptional corepressor limit bone formation in vivo. We also identify significant gaps in our mechanistic understanding of their interaction. Improved understanding of how these proteins regulate osteoclastogenesis and how their interactions are regulated may provide important new insights into physiological and pathological bone resorption.

## Supporting Information

S1 Fig Analysis of *Hdac7* ^*flox*^/*Hdac7* ^*flox*^ *; c-fms-Cre* osteoclast cultures. (TIF)

S1 Table Sequence of primers used for qRT-PCR (DOCX)
