# Supplementary figures and images for: Deletion of Histone Deacetylase 7 in Osteoclasts Decreases Bone Mass in Mice by Interactions with MITF
Source: PLoS One. 2015 Apr 15;10(4):e0123843. doi: 10.1371/journal.pone.0123843 (PMC4398560; doi:10.1371/journal.pone.0123843)

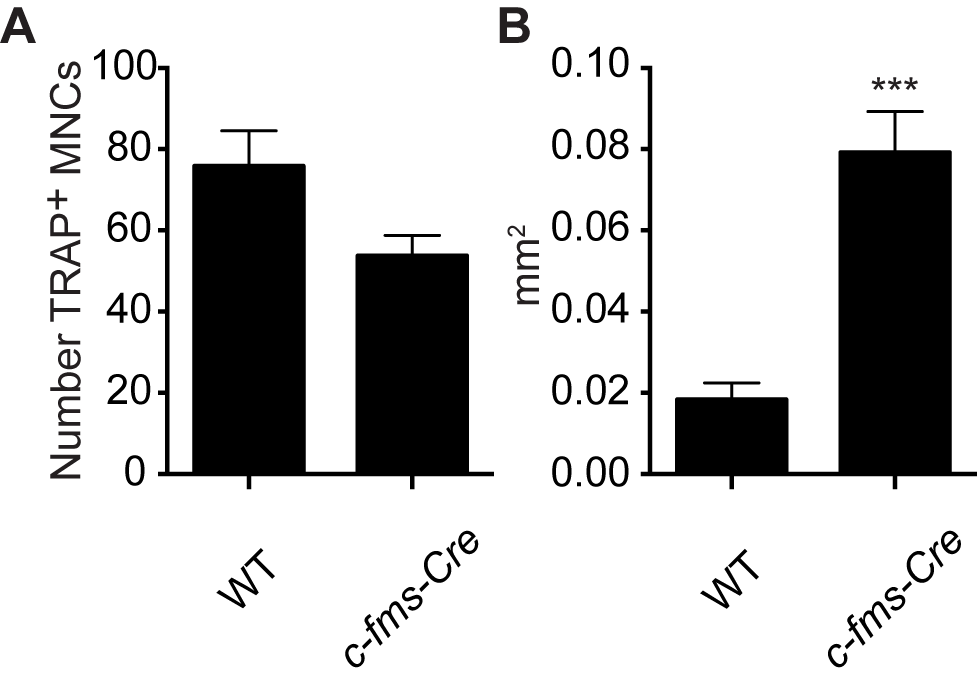

Supplement: S1 Fig — (TIF) [file pone.0123843.s001.tif]
