# Supplementary material for: Deletion of Histone Deacetylase 7 in Osteoclasts Decreases Bone Mass in Mice by Interactions with MITF
Source: PLoS One. 2015 Apr 15;10(4):e0123843. doi: 10.1371/journal.pone.0123843 (PMC4398560; doi:10.1371/journal.pone.0123843)
Supplement: S1 Table — (DOCX) [file pone.0123843.s002.docx]

**Table S1. Sequence of primers used for qRT-PCR**

| **Gene** | **Forward/Reverse Primer** | **Sequence 5’🡪3’** |
| --- | --- | --- |
| *Ctsk* | F | AGGGAAGCAAGCACTGGATA |
|  | R | GCTGGCTGGAATCACATCTT |
| *c-Fos* | F | TGGCACTAGAGACGGACAGA |
|  | R | TCCTACTACCATTCCCCAGC |
| *Dcstamp* | F | CAGACTCCCAAATGCTGGAT |
|  | R | CTTGTGGAGGAACCTAAGCG |
| *Hdac7* | F | CAGAACTCTTGAGCCCTTGG |
|  | R | GGATTCTTGCGTCTCTCCAG |
| *L4* | F | CCTTCTCTGGAACAACCTTCTCG |
|  | R | AAGATGATGAACACCGACCTTAGC |
| *Mitf* | F | GCTGGACAGGAGTTGCTGAT |
|  | R | GAAATTTTGGGCTTGATGGA |
| *Nfatc1* | F | TCATCCTGTCCAACACCAAA |
|  | R | TCACCCTGGTGTTCTTCCTC |
| *Oscar* | F | TCATCTGCTTGGGCATCATA |
|  | R | ACAAGCCTGACAGTGTGGTG |
| *Atp6v0d2* | F | TCAGATCTCTTCAAGGCTGTGCTG |
|  | R | GTGCCAAATGAGTTCAGAGTGATG |
